# Will web-based research suffice when collecting U.S. school district policies? The case of physical education and school-based nutrition policies

**DOI:** 10.1186/1479-5868-5-64

**Published:** 2008-12-10

**Authors:** Jamie F Chriqui, Michael Tynan, Tanya Agurs-Collins, Louise C Mâsse

**Affiliations:** 1 University of Illinois at Chicago, Institute for Health Research and Policy, 1747 West Roosevelt Road, M/C 275, Chicago, Illinois 60608, USA; 2 The MayaTech Corporation, 1100 Wayne Avenue, Suite 900, Silver Spring, Maryland 20910, USA; 3 Atlanta, Georgia 30324, USA; 4 National Cancer Institute, 6130 Executive Boulevard, Executive Plaza, Room 4076, Bethesda, MD 20852, USA; 5 University of British Columbia, Department of Pediatrics, CCHR Centre for Community Child Health Research, L408-4480 Oak Street, Vancouver, BC V6H 3V4, Canada

## Abstract

**Background:**

Recognizing the growing childhood overweight problem, a number of school-based strategies, including policy approaches, have been proposed and are being implemented to address the problem considering the amount of time children spend in schools. This paper describes the results of a pilot study that tested approaches to collecting U.S. school district policy information regarding physical education and nutrition requirements that can inform efforts by policy makers, researchers, advocates and others interested in collecting school district-level obesity-related policies that are typically not systematically available from a "one stop" source.

**Methods:**

Sixty local school districts representing six states were selected for conducting the district policy research, with larger, urban school districts over-sampled to facilitate collection of policies from districts representing a larger proportion of the public school population in each study state. The six states within which the pilot districts were located were chosen based on the variability in their physical education and school-based nutrition policy and geographic and demographic diversity. Web research and a mail canvass of the study districts was conducted between January and May 2006 to obtain all relevant policies. An additional field collection effort was conducted in a sample of districts located in three study states to test the extent to which field collection would yield additional information.

**Results:**

Policies were obtained from 40 (67%) of the 60 districts, with policies retrieved via both Web and mail canvass methods in 16 (27%) of the districts, and were confirmed to not exist in 10 (17%) of the districts. Policies were more likely to be retrieved from larger, urban districts, whereas the smallest districts had no policies available on the Web. In no instances were exactly the same policies retrieved from the two sources. Physical education policies were slightly more prevalent than nutrition policies.

**Conclusion:**

Collection of U.S. local school district policies requires a multi-pronged approach. Web research and mail canvasses will likely yield different types of policy information. Given the variance in district-level Web site presence, researchers and others interested in obtaining district physical education and nutrition-related policies should consider supplementing Web research with more direct methods.

## Background

Childhood obesity is a global problem, which until recently, predominantly affected developed countries. However, obesity is now on the rise in less developed countries as well.[1] In the U.S., childhood obesity is a significant and increasing problem with recent data indicating that in 2003–2004, over nine million U.S. children ages 6–19-or three times the proportion in 1980-had a body mass index in the 95^th ^percentile or higher. [2-4] Furthermore, a recent reanalysis of the NHANES 2003–2004 survey data using the International Obesity Task Force (IOTF) cut-offs suggests that U.S. children are experiencing a higher prevalence of overweight and obesity when compared to all industrialized countries.[5] As childhood obesity greatly impacts the global burden of chronic diseases and disabilities, it is crucial that global strategies are developed to curtail the rise in childhood obesity.[1]

Recognizing that obesity is caused by a combination of genetic, metabolic, behavioral, cultural, environmental, and socioeconomic factors,[6,7] a number of U.S. national organizations, including the Institute of Medicine (IOM), the National Alliance for Nutrition and Activity, the Clinton Foundation, the Alliance for a Healthier Generation, and the School Nutrition Association have developed strategies to address the problems of childhood obesity and overweight at the school, school district, community, municipal, state, and federal levels. Recently, the IOM and the World Health Organization (WHO) issued a call to governments, industry, communities, schools, and families to "identify, implement, evaluate and disseminate effective policies and interventions that support childhood obesity goals."[7,8]

Public policy approaches to addressing the obesity problem are gaining traction across the globe. In the U.S., reviews of state laws related to school-based nutrition and physical education (PE) indicate that, in many cases, state policies have created an enabling framework within which local school districts are able to develop their own policy provisions. [9-13] Globally, public policy strategies that potentially affect the obesity epidemic may relate to the school environment (e.g., PE time requirements or content standards); the community environment (e.g., land use management, density of food outlets); marketing and promotion practices (e.g., policies limiting advertising of unhealthy products); the retail environment (e.g., food taxation, nutrition labeling, and trans fat bans); and many others.[8,14-20] For example, across all levels of government in Australia, a systematic review of policies that influence the food and physical activity environments including the school was conducted to identify policy gaps, barriers, and opportunities for obesity prevention.[14]

As countries focus on environmental and policy strategies as possible precursors to initiating and sustaining obesity-related changes at all levels of the broader social system, it is important to be able to characterize what policies are in place to address this issue.[21] In the U.S., federal and state laws are readily available electronically via the Internet and from commercial legal service providers such as LEXIS-NEXIS and Westlaw. Likewise, many municipal and county level ordinances/codes are available on the Internet from municipal code publishers such as the Municipal Code Corporation, American Legal Publishing Corporation, General Code Publishers, Sterling Codifiers, Inc., and others. However, there is not a systematic, publicly-accessible centralized source that exists for obtaining copies of school district policies (i.e., policies, procedures, and regulations adopted by local education agencies, school boards, and local departments of education) throughout the U.S. In some states, a state agency or state association of school boards collects school district policy information but access is often limited to districts in the state and, in some cases, limited to member-only access. The Prevention Institute provides the ENACT Local Policy Database which catalogues promising local policy practices in physical activity and nutrition, but does not contain a census of all documents across all districts in the U.S.[22] Thus, policy makers, evaluation researchers, and other practitioners who are interested in obtaining copies of local school district policies across states are limited to common data collection techniques including mail-based canvasses, Web-based research, and field-based data collection.

To this end, the purpose of this study was to test the feasibility of collecting PE and school-based nutrition policies from local school district jurisdictions in the U.S. using a multi-pronged strategy involving Web-based research followed by telephone and mail follow-up. Results are based on a pilot study conducted in 60 U.S. school districts located in six states to explore the strategies and challenges associated with collecting school district policies. The information presented herein will provide useful insights for facilitating future policy collection in these areas, and will provide a methodology which can be applied to any country where school-based nutrition and PE policies have been decentralized and are not systematically catalogued into a publicly accessible central database. This study also will provide useful insights for: (1) ongoing efforts in the U.S. to collect wellness policies which were mandated by Congress under P.L. 108–265, since the methodology for district-level policy collection should not vary by content area and, in many cases, the policies collected for this study are now subsumed in U.S. school district wellness policies; and (2) for efforts to collect other types of obesity-related policies from local government jurisdictions (e.g., counties and municipalities in the U.S.).

## Methods

### Subjects

The pilot study was comprised of a sample of 60 school districts located within six states – California, Georgia, Illinois, Massachusetts, New York, and Texas – that represented both a variety of policy approaches as well as geographic and demographic variance. The six study states were identified based on a review of the state PE and school-based nutrition policies to assess policy variance [9,10] and state-level demographic data from the Census. The policy/geographic variances of the selected states are presented in Table 1.

**Table 1:** Local pilot state selection

*State*	State Policy Extensiveness*	*Geographic Area*
California	High	West
Georgia	Middle	Southeast
Illinois	Middle/Low	Midwest
Massachusetts	Low	Northeast (township oriented)
New York	High	Northeast (not township oriented)
Texas	Middle	South Central

* The classification of state policy extensiveness was based on a review of state physical education and school-based nutrition policies as of December 31, 2003 published in a supplement to the American Journal of Preventive Medicine.[9,10] These data were the only state policy classification data available at the time of the local sample selection. "High" state policy extensiveness indicated that the state law contained more in-depth policy provisions in the areas of PE and school-based nutrition. "Low" state policy extensiveness indicated that the state law contained little, if any, details relative to state guidelines governing PE and/or school-based nutrition issues.

The universe of possible school districts from which to draw the study sample was obtained from the Census of Governments for 2002 [via the Governments Integrated Directory (GID) Public Use Files)]. Data were retrieved from the Census Bureau Web site on November 8, 2005, and the data included the enrollment figures based on the 2002 Census data. Based on these data, 10 school districts within each of the study states were selected by stratifying the school districts within each state by quartile and selecting the largest districts within each quartile as follows: quartile 1 (largest) – top five districts based on enrollment size, quartile 2 – top two districts based on enrollment size, quartile 3 – top two districts based on enrollment size, and quartile 4 – top district based on enrollment size. Considering that this was a pilot study, focus was placed on selecting policies from school districts that represented a larger proportion of the public school population in each study state, so the largest (urban) districts were over-sampled in comparison to the smaller districts.

### Procedure

The school district policies were collected between January and May 2006 via Web research and a mail solicitation requesting hard or electronic copies of the district policies. For purposes of this study, "policy" was defined as the formal proscriptions developed by local school boards (often called "Board Policy") and any associated implementation regulations or procedures. Such formal, board policies would be akin to the formal laws developed by local, state or national legislative bodies. Informal policies such as memoranda of understanding, question and answer documents, and interpretation memoranda were excluded. In three of the study states, policies also were retrieved in a sub-sample of the districts using field-based policy collection (i.e., site visit) methods. In only one instance did the site visits yield additional policy documents beyond that obtained via the mail or Web-based policy collection strategies. This instance indicated the need to expand the definition of "policy" to include "procedure" documents as developed by the districts because these documents carry the force of law in these districts and are implemented as such.

#### Web research

##### Identifying district policies

With the exception of one district, Web sites were identified for each school district. Two primary types of policy documents were garnered from the school district Web sites – policy manuals and other policy-relevant documents. The policy manuals reflect the codification of the school policies established by the school district or board of education for the district; these manuals are akin to a state legislature enacting laws that are codified in the state statutes.[23] Other policies collected via the Web sites included policies originating from district superintendents' offices in the form of bulletins, memos, regulations, procedures, and other regulatory-like documents.

##### Retrieving relevant policies

Initial reviews of each Web site involved searching for the district's policy manual which typically was found on the Web page for the district's Board of Education. The superintendent's Web page also was reviewed to identify additional policies relevant to PE and school nutrition. If relevant policies were not found in the policy manual or the superintendent's office, the Web pages for other offices such as the food service office and the curriculum/learning office were searched to retrieve policies. Each Web site was also queried by keyword, including but not limited to the following: *policy*, *nutrition*, *physical education*, *health*, *vending*, *lunch*, and *obesity*.

##### Verifying complete retrieval

All initial searches of the district Web sites were conducted by two researchers and following completion of the Web site searches, a third researcher reviewed each districts' site to confirm complete capture of all available and possibly relevant information.

#### Mail solicitation

Dillman's tailored design method [24] was employed to conduct a mail-based canvass of officials in each of the 60 study districts to request hard or electronic copies of their policies related to nutrition and PE. The mail canvass involved identifying the appropriate contact(s) within each school district to whom requests for policy documents should be sent, sending an initial mail request, and conducting two follow-up requests.

##### Identifying relevant contacts

Identifying Relevant Contacts. A snowball sampling method [25] was used to determine the relevant contact(s) in each school district responsible for providing copies of the district policies. The primary challenge at this stage of the process was the varied approaches to governing and organizing school districts within each state.[26] Such jurisdictional variation needs to be considered when conducting multi-state studies since the governing (i.e., policy) authority will rest with different jurisdictions based on the state. For example, many school districts in New England are organized by town or township with policies developed at the township level even though the towns are located within counties in the state; while school districts in North Carolina, Tennessee, and Virginia are organized by county and policy making in these states is generally done at the county level. [26]

Data obtained from Internet search engines (e.g., ) and from the National Center for Education Statistics' (NCES) district search tool  were used to determine initial school district contact information. If the school district's Web site indicated an organizational hierarchy, school flow chart, or directory, an effort was made to identify possibly relevant contacts responsible for providing copies of relevant policies. In instances where a relevant contact(s) could not be identified, the superintendent's office (or the equivalent) was contacted. School districts that offered policy information online tended to defer to the district Web site rather than identifying a point of contact, stating that the Web site provided access to all of their policies. In some situations, multiple contacts were identified (e.g., food service director and an administrator in charge of health or physical activity). For example, in New York City, project staff could not identify one valid contact point through telephone calls. As a result, six letters were mailed to various contacts, including the Chief of Staff, Policy Advisor, Staff Physicians, and the Food Service Director; all contact points that were obtained from the school district's Web site.

##### Requesting policy information

Following Dillman's method,[24] a five-step process was employed for requesting the school district policies from the identified contacts: (1) initial contact letter, (2) follow-up phone call, (3) follow-up letter, (4) verification letter, and (5) thank you letter.

##### Initial contact letter

Initial contact letters were sent via FedEx to the confirmed points of contact during the month of January 2006, detailing the information sought and indicating the process for submitting policies. This letter included a list of policy topics of interest and requested that contacts provide as much policy information as possible so research staff could determine what information was relevant for consistency purposes. In many cases, the initial letter spurred a return telephone call from school districts. Their responses ranged from declining to participate to responding that the policies would be mailed soon.

##### Follow-up telephone call

The purpose of this first follow-up contact was to: (1) touch base with the school district contact(s) that had not yet sent their policies, (2) verify that they had received and understood the initial request, (3) determine if they were in the process of responding to the request, and (4) to respond to any questions they had. The follow-up telephone calls occurred approximately 2 to 2 1/2 weeks after the initial letter, with most of the contact attempts occurring between the first two weeks of February 2006. Contacts that already had declined to participate or already had sent policies were not contacted by telephone.

##### Follow-up letter

Approximately one week after the follow-up telephone call, a follow-up letter, similar to the initial letter, was sent via FedEx to the non-responding contacts.

##### Verification letter

During the initial contact calls, four school districts directed project staff to their online policies and indicated they would provide further assistance by verifying the information obtained from the school district Web site. Although the school district Web sites were already searched under the Internet-based policy information collection strategy discussed above, an additional Web site search was conducted for these four school districts as a way to further test the Internet collection strategy. The Web site searches were followed by individualized letters listing specific policy topics that could not be located on the Web and asking the contacts to confirm that no policies were indeed available for these specific topic areas.

##### Thank you letter

Finally, a thank-you letter was sent to respondent contacts to indicate receipt of the policies and conclusion of the school district's participation in the study.

### Data Analysis

Descriptive statistics were computed using SPSS v. 14.0.1 to assess the policy retrieval rate for the Web collection and policy response rate for the mail canvass. Independent sample t-tests were conducted to assess the difference in sample means for policy retrieval based on the policy collection source (i.e., Web or mail), state, and district size.

## Results

**Figure 1:**
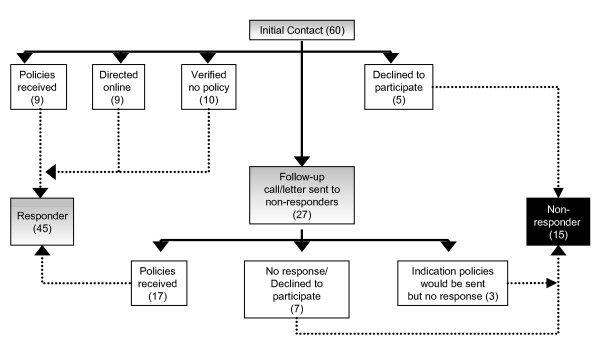
Mail-based canvass response pattern.

Forty-five of the 60 districts (75%) responded to the mail canvass; of these, 26 districts submitted policies through either the initial mailing or follow-up contacts, 10 districts confirmed that policies did not exist, and nine districts indicated that their policies were available on-line (see Figure 1). In three districts (5%), respondents indicated that they would submit the policies; however, they were never received and, therefore, these districts were included with the non-responders (n = 15 districts; 25%). Policies also were retrieved from 30 (50%) of the districts' Web sites. Ultimately, relevant policies were obtained from 40 (67%) of the 60 districts (regardless of source) and confirmed to not exist in 10 districts (17%). In 16 of the 40 districts for which policies were obtained, they were retrieved via both Web and mail methods (see Table 2).

**Table 2:** District policy retrieval rates by state, quartile, policy collection method, and topic

***State***	***Districts for which any policies were retrieved (N = 10/state)***	Districts for which policies were retrieved by quartile*				Districts for which policies were retrieved by policy collection method** (N = 10/state)			***Districts for which policies were retrieved by topic (N = 10/state)***	
		
		**1 (N = 5/state)**	**2 (N = 2/state)**	**3 (N = 2/state)**	**4 (N = 1/state)**	**Mail**	**Web**	**Both**	**Physical Education**	**Nutrition**
California	7	4	1	2	0	7	5	5	7	7
Georgia	9	5	1	2	1	6	6	3	9	9
Illinois	4	4	0	0	0	2	4	2	4	4
Massachusetts	5	4	0	1	0	2	5	2	5	4
New York	7	5	1	0	1	5	4	2	6	3
Texas	8	5	1	2	0	4	6	2	8	8

**Total (%)**	**40/60** **(66.7%)**	**27/30** **(90.0%)**	**4/12** **(33.3%)**	**7/12** **(58.3%)**	**2/6** **(33.3%)**	**26/60** **(43.3%)**	**30/60** **(50.0%)**	**16/60** **(26.7%)**	**39/60** **(65.0%)**	**35/60** **(58.3%)**

*Quartile 1 = largest districts; quartile 4 = smallest districts.

**Data are not mutually exclusive.

Policies were most likely to be retrieved from the largest, urban (quartile 1) districts (90% had policies). These districts also were significantly more likely than smaller districts to have relevant policies available on their Web sites (t = 6.812, p < .001) and for policies to be obtained via both mail and Web methods (t = 3.099, p < .01). On the other hand, policies were least likely to be retrieved from the smallest (quartile 4) districts, with none of the quartile 4 districts having relevant policies available on their Web site and only two of the six districts in this quartile submitting policies in response to the mail request. Interestingly, the quartile 3 districts were more likely (although not significantly) than the quartile 2 districts to submit policies and/or to make policies available on their Web site.

Policy retrieval also varied by state. Relevant policies were more prevalent in school districts in Georgia (9/10 districts), Texas (8/10 districts), and California and New York (7/10 districts each). Policies were only retrieved from four districts in Illinois and five districts in Massachusetts, with only two districts in each of these states submitting policies via the mail request (although one of the Illinois districts submitted a mission statement which was not deemed relevant but was counted as making a submission for response purposes). Policies were obtained from the Web sites for at least four of the 10 districts in each of the six study states.

The policies retrieved via the two collection methods varied by source. In two districts, the policies retrieved via each source were completely different. In 13 districts, some of the same policies were retrieved from both sources, and in 15 districts some of the policies were different between the two sources. In no instance were all of the same policies retrieved from both the mail and Web sources.

PE policies were obtained from 39 of the 40 districts for which relevant policies were received; nutrition policies were obtained from 35 of the 40 districts. More than three-quarters of the districts for which relevant PE or nutrition policies were obtained used both methods with very little overlap in the actual policies retrieved by each method. As Table 3 indicates, the prevalence of PE and nutrition-related provisions contained within the policies varied greatly, more heavily emphasizing specific topics such as instruction time, staffing requirements, and minimum PE content standards in the PE topic area and emphasizing food and beverage sales through a la carte, vending, and other venues as well as nutrition education in the nutrition topic area. PE and nutrition-related provisions were found in policies retrieved from each of the data collection methods examined for this study.

**Table 3:** Local policy topics by collection method

*Topic*	*Method*			*Total (N = 60)*	
	Mail	Web	Both	n	%
**Physical Education**					

Time requirements	7	13	12	32	53.3
Staffing requirements	3	5	7	15	25.0
Standards for PE	3	13	11	27	45.0
Assessment of health-related fitness	1	2	1	4	6.7
Recess	2	1	4	7	11.7

**Nutrition**					

A la carte F&B sales	6	3	10	19	31.7
Vending machines	7	7	13	27	45.0
Other competitive food sales	7	5	13	25	41.7
School meal environment	0	0	1	1	1.7
Reimbursable school meals	0	0	2	2	3.3
Food service director requirements	2	1	2	5	8.3
Nutrition education	2	12	10	24	40.0
Advertising	0	1	0	1	1.7
Pricing	0	0	0	0	0
Health advisory committee	2	7	3	12	20.0
BMI screening	1	0	2	3	5.0

Wellness policies (which were mandated as part of the *Child Nutrition and WIC Reauthorization Act of 2004, P.L. 108–265 *as of the first day of the school year following June 30, 2006) were submitted by 13 districts in response to the mail canvass; no wellness policies were retrieved from the district web sites. This finding was not surprising given that the local wellness policy mandate did not take effect until after the study reference date; however, it was interesting that none of the districts included in the sample had wellness policies posted on their web sites during the months leading up to the federal mandate deadline.

## Discussion

In the U.S., local school district policies are not systematically available via a single, electronic source, much less via individual, statewide sources. Thus, collection of local school district policy information is limited to common data collection techniques, including mail-based surveys, Web-based research, and field-based data collection. As indicated above, the mail and Web-based strategies may yield different results, indicating that a combination of approaches is necessary for any effort seeking to obtain as much policy information as possible from a given district. Or, at a minimum, indicating the need to verify policies collected solely from the Web to ensure complete capture. Although we conducted a limited field-based research canvass in a few districts in three study states, we do not have a sense as to the completeness of the policy information obtained from each school district (regardless of method). Future efforts would be well-served to employ some type of verification process (phone- or electronic-mail based) to address this concern.

Since all school districts participating in the National School Lunch Program (NSLP) or federal Child Nutrition Programs in the U.S. were required to have wellness policies that include, among other things, nutrition education goals, guidelines regarding the sale of all food and beverages sold during the school day, an assurance that reimbursable school meals meet Federal requirements, and physical activity goals by the first day of the 2006/2007 school year, it is possible that the findings of this study would have differed if it was conducted after the wellness policy mandate became effective. However, a review of sample wellness policies obtained through this study and additional wellness policies collected by members of the study team since the time of this study, indicated that many of the wellness policies actually cross-reference other district policies (e.g., vending machine policies, curricula requirements, etc.) that are not readily incorporated into the actual wellness policies. As such, researchers and other interested parties will still need to develop a process for systematically capturing all relevant policies, and not just the wellness policies. Thus, we speculate that many of the challenges presented in this study will still apply; however, it is expected that initial collection of the wellness policies will be fairly accessible via either the Web or a mail/telephone request for the districts participating in the NSLP or Child Nutrition Programs. Yet, the ability to collect even the wellness policies *solely *by Web research will likely be difficult given variability in district resources and, as a result, a multi-pronged policy collection strategy should still be considered.

Bearing this in mind, the response rate to the mail-based canvass was somewhat encouraging – 45 (75%) of the districts included in the study sample responded to the request to submit policies (either affirmatively or verifying that a policy did not exist). This response rate was higher than other documented PE/nutrition-related school district level collection efforts [conducted by the School Nutrition Association (SNA) and the United States Government Accountability Office (GAO)]. SNA conducted an online survey of all school nutrition directors in the Association's membership database (N = 4,850) and received a 14 percent response rate.[27] At the same time, the GAO conducted two Web-based surveys – one of the school food authority directors and the other of school principals – between October 2005 and February 2006 regarding competitive food sales in school environments. The GAO surveys yielded response rates of 70 and 65 percent, respectively.[28] While both the SNA and the GAO surveys were entirely Web-based and therefore did not employ comparable methodologies to our study approach, they were the only similar types of efforts that attempted to collect school district-level nutrition-related information that we were able to identify. Acknowledging this limitation, their response rates were lower than the response rates for the study described herein.

At the same time, due to the complexity of multi-layered organizational structures within certain school districts, it is possible that the respective organizational units within these districts were unclear as to which unit was responsible for responding to the request and, therefore, they did not respond or responded only to certain components of the request. As a result, the policy collection for these districts may be incomplete or missing. Further, the review of the policies was limited solely to the policies that were available via the primary data collection strategies employed for this study – Web-based research with telephone follow-up and a mail-based canvass. A non-systematic and random review of a few districts' Web sites following the data collection time period revealed that a number of districts had posted new information on their Web site following the study data collection period. Thus, the policies reviewed for this study were solely reflective of the policies submitted by the districts during the study time frame. Researchers and other interested parties are encouraged to recognize the fact that any policy collection is always going to be a "moving target" for two primary reasons. First, policies can change at any minute on any given day, which is the reality of policy research. Secondly, Web-based material is also subject to constant updates and changes. As such, it is important for the field to recognize these limitations and to clearly identify the study reference date/policy collection window so that the audience for their work can understand the results in context of the time period during which the policies were collected. An additional important limitation is that since this study solely focused on testing methods for *collecting *school district policies, we are unable to report on implementation or impact of these policies on student-level behaviors. This is a critical next step for future research efforts.

Finally, although the methodology was only tested in the U.S. and was limited to school district policies, it can be used in other countries and applied to other types of local policy collection (e.g., counties and municipalities in the U.S.). The methodology will be most useful in countries where: (1) school-based nutrition and PE policies are enacted at the local government level (i.e., not centralized), (2) the policy information is not systematically catalogued in a common database, (3) the information is publicly available, and (4) where the information is commonly posted on the Web. Likewise, a similar methodology could be applied for efforts to collect other types of school district policies or other types of local policies such as those related to the community environment (e.g., land use management, density of food outlets); marketing and promotion (e.g., policies limiting advertising of unhealthy products); and the retail environment (e.g., food taxation, nutrition labeling, and trans fat bans).[8,14-20] In the U.S., the major difference would be that access to county and municipal laws for many local governments is available through one of several municipal code publishers including the Municipal Code Corporation , American Legal Publishing Corporation , General Code Publishers , Sterling Codifiers, Inc. , and others. Thus, a review of each of these sites for the community(ies) of interest would be a critical first step in the research process. In cases where such information is not available through a municipal code publisher, then researchers would be advised to follow the steps presented herein, as they will also apply. Notably, should a mail or telephone follow-up be required for these non-school district policies, the most likely starting point would be the county or municipality's clerks' office for the respective community (rather than the school district office). Prior and ongoing research conducted by the study authors related to county/municipal laws suggests that a similar pattern will be found in terms of policy availability on-line; in other words, larger, more resource-laden communities will be more likely than smaller communities to have their laws available on-line either through a municipal code publisher or on their own website.

## Conclusion

This study revealed that it is necessary to employ a multi-pronged approach to collecting PE and school-based nutrition-related policies from local school districts in the U.S. Although the study described herein cannot be generalized to all districts or other countries, it points to a number of factors that must be considered when seeking to obtain such information. Given the movement toward recognizing policy interventions as one strategy to curtail the global childhood obesity problem [8-10,12-17,20,29], new technologies and strategies will be necessary to stay abreast of the variety of policy approaches that various jurisdictions are taking in this regard. Clearly, web-based technologies will likely be the most prominent sources of this information, however, technology cannot take the place of local officials who know the content of the local governments' policies and who would be best positioned to point interested individuals to the most relevant policies adopted in the given area.

## Competing interests

Dr. Jamie Chriqui and Michael Tynan's contributions on this manuscript were conducted as part of a contract from the National Cancer Institute (NCI) to The MayaTech Corporation. Dr. Tanya Agurs-Collins served as the NCI Project Officer. Dr. Louise Mâsse was the initial NCI Project Officer and, subsequently, as a consultant to NCI on this project. NCI supported the development of this manuscript through contract numbers N02-PC-444006 and 263-MQ-515012 to The MayaTech Corporation.

## Authors' contributions

JC led this study and the development and drafting of this manuscript. MT led the data collection reported on herein and contributed to the manuscript development. TAC and LM helped to conceptualize the study and contributed to the manuscript development and review.
